# Supplementary material for: The Role of Physical Therapies in Wound Healing and Assisted Scarring
Source: Int J Mol Sci. 2023 Apr 19;24(8):7487. doi: 10.3390/ijms24087487 (PMC10144139; doi:10.3390/ijms24087487)
Supplement: Supplementary file 1 [file ijms-24-07487-s001.zip › ijms-2321712-supplementary.pdf]

Supplementary Figure

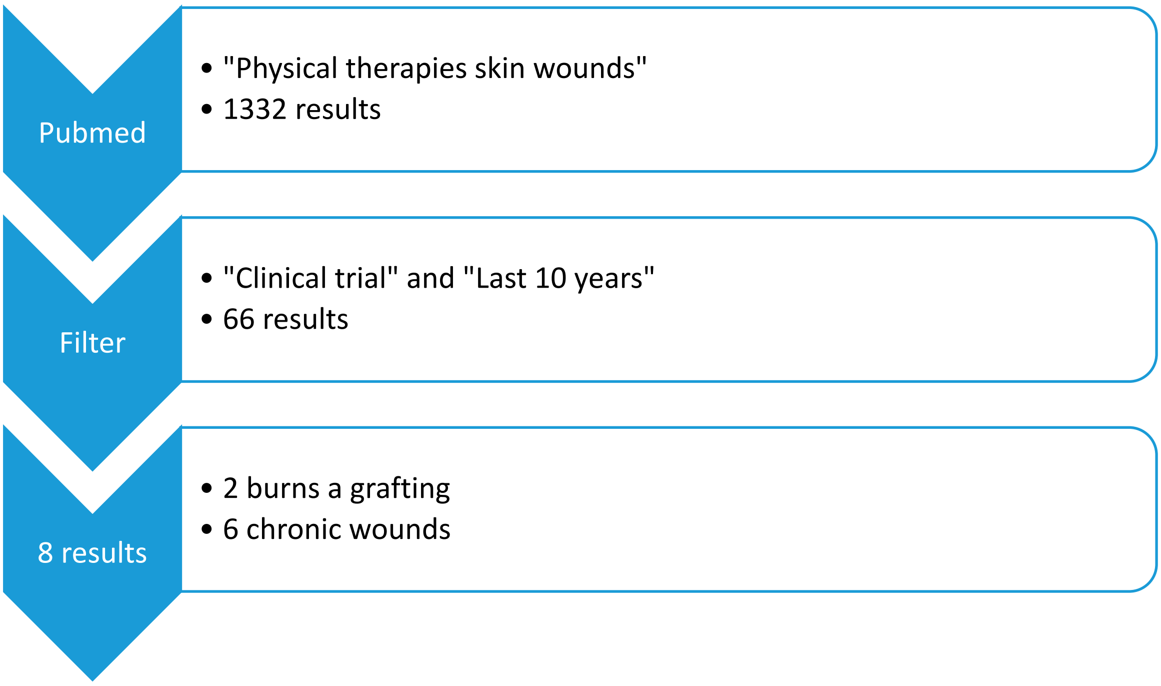

Figure S1. Clinical trial published of intervention with physical therapies in wound healing in pubmed.
